# Effect of Arbidol (Umifenovir) on COVID-19: a randomized controlled trial

**DOI:** 10.1186/s12879-020-05698-w

**Published:** 2020-12-14

**Authors:** Marzieh Nojomi, Zeynab Yassin, Hossein Keyvani, Mahin Jamshidi Makiani, Maryam Roham, Azadeh Laali, Nasir Dehghan, Mehrnaz Navaei, Mitra Ranjbar

**Affiliations:** 1 Preventive Medicine & Public Health Research Center, Psychosocial Health Research Institute, Department of Community and Family Medicine, School of Medicine, Iran University of Medical Sciences, Tehran, Iran; 2 Antimicrobial Resistance Research Center, Department of Infectious Disease, School of Medicine, Hazrat-e Rasool General Hospital, Iran University of Medical Sciences, Tehran, Iran; 3 Department of Virology, School of Medicine, Gastrointestinal and Liver Disease Research Center, Iran University of Medical Sciences, Tehran, Iran; 4 Antimicrobial Resistance Research Center, Iran University of Medical Sciences, Tehran, Iran; 5 Department of Infectious Disease, School of Medicine, Firoozgar General Hospital, Iran University of Medical Sciences, Tehran, Iran; 6 Department of Community and Family Medicine, School of Medicine, Iran University of Medical Sciences, Tehran, Iran; 7 Department of Infectious Disease, School of Medicine, Iran University of Medical Sciences, Tehran, Iran

**Keywords:** Arbidol, Corona virus, COVID-19, Efficacy

## Abstract

**Background:**

Treatment of patients with COVID-19 has included supportive care to mainly relief symptoms of the disease. Although World Health Organization (WHO) has not recommended any effective treatments for COVID-19, there are some reports about use of antiviral drugs. The aim of this study is to determine the effect of Arbidol (ARB) on COVID-19 disease.

**Methods:**

Using an open-label randomized controlled trial, we examined the efficacy of ARB in patients with COVID-19 in a teaching hospital. One hundred eligible patients with diagnosis of COVID-19 were recruited in the study and assigned randomly to two groups of either hydroxychloroquine followed by KALETRA (Lopinavir/ritonavir) or hydroxychloroquine followed by ARB. The primary outcome was hospitalization duration and clinical improvement 7 days after admission. The criteria of improvement were relief of cough, dyspnea, and fever. Time to relief from fever was also assessed across the two groups. Without any dropouts, 100 patients were entered into the study for the final analysis at significance level of 0.05.

**Results:**

The mean age of patients was 56.6 (17.8) years and 56.2 (14.8) years in ARB and KALETRA groups, respectively. Majority of patients were male across two groups (66 and 54%). The duration of hospitalization in ARB group was significantly less than KALETRA arm (7.2 versus 9.6 days; *P* = 0.02). Time to relief fever was almost similar across two groups (2.7 versus 3.1 days in ARB and KALETRA arms, respectively). Peripheral oxygen saturation rate was significantly different after 7 days of admission across two groups (94% versus 92% in ARB and KALETRA groups respectively) (*P* = 0.02). Based on multiple linear regression analysis, IHD, Na level, and oxygen saturation at the time of admission and type of therapy were the independent adjusted variables that determined the duration of hospitalization in patients with COVID-19.

**Conclusion:**

Our findings showed that Arbidol, compared to KALETRA, significantly contributes to clinical and laboratory improvements, including peripheral oxygen saturation, requiring ICU admissions, duration of hospitalization, chest CT involvements, WBC, and ESR. We suggest further studies on ARB against COVID-19 using larger sample size and multicenter design.

**Trial registration:**

IRCT20180725040596N2 on 18 April 2020.

## Background

Coronavirus disease (COVID-19) is an infectious disease caused by a new coronavirus named SARS-COV-2. Majority of people infected by this virus will experience mild to moderate symptoms of respiratory illness. This virus can be transmitted by droplets when an infected person sneezes or coughs [1].

Since first cases of COVOD-19 were reported in December 2019 from Wuhan in China, more than 42 million cases of this disease have been reported globally leading to more than 1,100,000 deaths [2]. COVID-19 has affected more than 180 countries and the problem is going to be worse due to there are no specific vaccines or treatments for COVID-19. World Health Organization (WHO) declared the outbreak of novel Corona virus a Public Health Emergency of International Concern, or PHEIC on January 2020 [3].

This is a severe problem for public health because the majority of infected people do not develop symptoms but could transmit the disease to the others during the incubation period [4].

There are currently no known effective therapy for infection with SARS-COV-2. Treatment of moderate to severe forms of COVID-19 treatment is generally necessary. Treatment of patients with COVID-19 is mainly supportive care to relief symptoms. Although WHO has not recommended any effective treatments for COVID-19, there are reports about the use of some antiviral drugs (oseltamivir, lopinavir/ritonavir), antibiotics, hydroxychloroquine and glucocorticoids for treatment in this patient population [4]. However, there are many ongoing clinical trials evaluating potential treatments for COVID-19.

The other drugs also were recommended as a possible therapeutic options for the COVOD-19 such as Remdesivir and Chloroquine phosphate [5, 6].

Recently Australian scientists have published a research indicating that Ivermectin, an approved anti-parasitic drug is highly effective against the COVID-19 virus when applied to an infected cell culture [7].

Arbidol (ARB) also known as Umifenovir is a Russian-made drug using for some enveloped and non-enveloped viruses. ARB is well known in Russia and China, and with a lesser extent in other countries but not in North America. Arbidol has been used against influenza A and B viruses, and recently against hepatitis C virus (HCV) [8, 9]. ARB can prevent contact and penetration of virus to host cells via avoiding the fusion of the virus lipid shell to the cell membrane. It has been shown that ARB could inhibit COVID-19 infection through interfering the release of SARS-CoV-2 from intracellular vesicles [10].

At present, there is not any potent and specific antiviral therapy or vaccine for SARS-COV-2. Therefore developing an effective drug for therapy or control of this disease is very critical option to control the COVID-19 outbreak.

The aim of current study was to determine the efficacy of ABD in treatment of COVID-19.

## Methods

### Design and participants

This open-label randomized controlled trial of efficacy of ARB against COVID-19 was conducted between 20 April and 18 June 2020 in a teaching hospital in Iran University of medical Sciences (IUMS) in Tehran, Iran. The current clinical trial was done in accordance with the principles of the Declaration of Helsinki and the International Conference on Harmonization–Good Clinical Practice guidelines. The study protocol was approved by the ethics committee of Iran University of Medical Sciences (IR.IUMS.RCT.1399.090) and registered in Iranian Registry of Clinical Trials (IRCT) with register number of IRCT20180725040596N2 on 18 April 2020 (URL: https://www.irct.ir/user/profile). The study protocol and reporting of results were adhered to Consolidated Standards for Reporting Trials (CONSORT).

Eligible patients were non-pregnant women and men aged 18 years or older with definite diagnosis of COVID-19 by Real Time RNA specific Reverse Transcriptase Polymerase Chain Reaction (RT-PCR) or computed tomography (CT) scan imaging (pneumonia), and oxygen saturation of 94% or less. The findings of CT were described as bilateral lung opacities and lobular and sub segmental areas of consolidation [11]. Patients were enrolled into the study from hospitalized patients who were admitted to the infectious diseases ward of Firoozgar teaching hospital. We considered a significance level of 0.05 and 80% power to detect a moderate difference (Standardized difference < 0.05) of hospitalization duration across two groups. Based on these criteria, the sample size was calculated as 50 per group [12].

Patients gave written informed consent according to regulations of ethics committee. We excluded participants who had a history of allergy to ARB class of drugs, abnormal liver or renal function, abnormal blood coagulation, prior use of ARB, women who were nursing or were pregnant, and patients with severe heart disease.

### Intervention

Participants were assigned to the intervention or control groups using blocked randomization method. Envelopes were prepared for unmasking randomization and allocation concealment. The random allocation procedure was performed by an independent staff of the hospital in which the RCT was run. The project manager and other colleagues of current study enrolled and assigned participants to the interventions.

In this trial patients with diagnosis of COVID-19 received either hydroxychloroquine (400 mg on first day) followed by 400 mg KALETRA (Lopinavir/ritonavir) BD or Hydroxychloroquine (400 mg BD on first day) followed by ARB (200 mg TDS) 7 to 14 days based on the severity of disease. The capsule of ARB (100 mg) was used in this trial.

Patients monitored daily for adverse events, vital signs, and changing of signs and symptoms. The current trial was monitored by research branch of the Food and Drug administration organization of Iran.

### Outcome measures

The research objective of this study was the assessment of potential superiority of ARB compared to KALETRA on management of patients with COVID-19. The primary outcome was duration of hospitalization and clinical improvement 7 days after admission. The criteria of improvement were relief of cough, dyspnea, and fever. Time to relieving fever was also assessed across the two groups.

The secondary outcomes were death during the 30 days of treatment, duration of hospitalization, changing laboratory tests during 7 days, changing of CT findings after 30 days, and the need for invasive mechanical ventilation.

We documented the age, gender, job, education status, underlying disease (history of diabetes, ischemic heart disease, hypertension, asthma,) and smoking status, as demographic variables. Fever, cough, dyspnea, nausea and vomiting, diarrhea, fatigue and weakness, loss of appetite, and taste were also documented at the first day of admission. The saturation of peripheral oxygen, C-reactive protein (CRP), complete blood cell count (CBC), erythrocyte sedimentation rate (ESR), aspartate transaminase (AST), alanine aminotransferase (ALT), white blood cell count (WBC), lymphocyte and neutrophil count, neutrophil lymphocyte ratio, total bilirubin, blood Na and K, creatinine and thyroid stimulating hormone (TSH) were measured at the first day of admission and 7 days after. The CT scan and chest X-ray were taken at the first day of admission and 30 days after. The PCR test was taken at admission and the time of discharge.

We considered the following criteria for discharge from hospital: Resolution of fever at least for 72 h and decreasing trend in LDH and CRP and lymphocytosis significant and having normal saturation of peripheral oxygen.

We categorized the patients to three groups based on severity of disease according to the CT scan and chest X-ray findings. Mild, moderate and severe categories were defined as involvement of base, less and more than 50% of the lung field respectively in CXR and CT scan.

### Statistical analysis

Data analysis was carried out using SPSS version 24 software (SPSS Inc., IL, and USA). The normality of data was evaluated using Kolmogorov–Smirnov (KS) test. Descriptive statistics including mean, frequency, and standard deviation (SD) were calculated for all numeric variables and expressed as mean ± SD. Chi-square test was used to compare the qualitative variables across two groups. For normally distributed variables, independent sample and paired *t*-test were used across groups and before-after analysis, respectively. We also used one way Analysis of Variance (ANOVA) to assess numeric variables across groups more than two category. Correlation analysis was used to evaluate the association between numeric variables. The *B* regression coefficients, with 95% confidence intervals (CI), were obtained using multiple linear regression analysis to assess the covariates associated with duration of hospitalization adjusted. All analyses were performed two-sided and significant level was considered at 0.05.

## Results

**Fig. 1:**
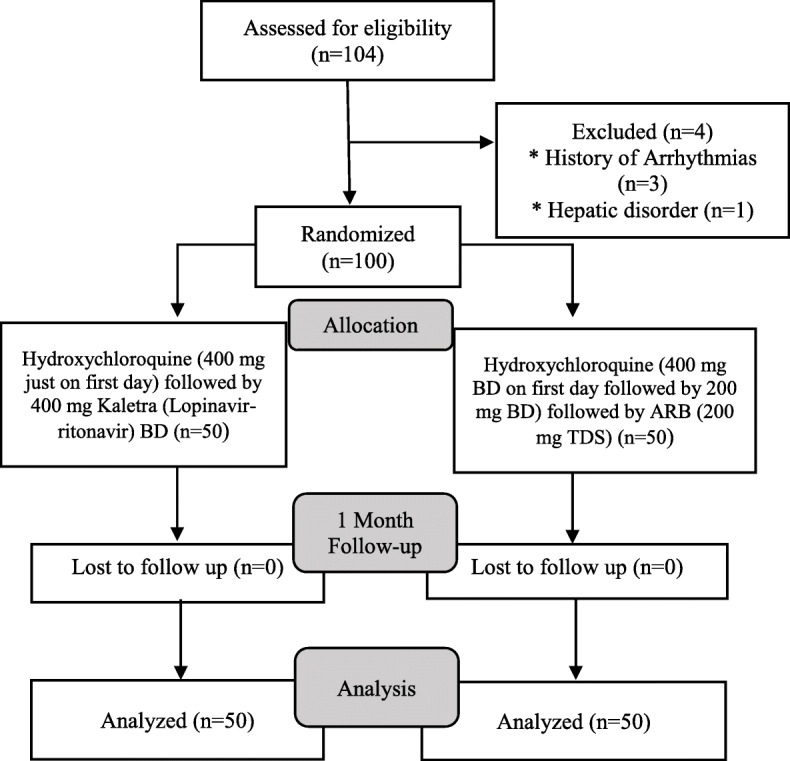
Consolidated standards of reporting trials (CONSORT) flow diagram

Of 104 recruited patients with COVID-19 admitted to the hospital between 20 April and 18 June 2020, four subjects were excluded due to contraindication of ARB use. The remaining one hundred patients who fulfilled inclusion criteria were assigned to the study and randomized to hydroxychloroquine plus KALETRA or hydroxychloroquine plus ARB. We did not have any lost to follow-up; therefore 50 patients per group were entered to final analysis (Fig. 1). The numeric data was adhered to normal distribution using KS test (*P* < 0.05).

Table 1 illustrates the demographic characteristics of the patients. The mean age of the patients was 56.6 (17.8) years and 56.2 (14.8) years in ARB and KALETRA groups, respectively. Majority of patients were male across two groups (66 and 54%). The percent of smokers in ARB group was significantly higher than KALETRA group (*P* = 0.01). About 40% of subjects reported history of contact with a patient with COVID-19 during 2 weeks before admission.

**Table 1:** Demographic Characteristics of the Patients by Treatment Groups

Characteristics^a^	All patients (***n*** = 100)	Arbidol group (***n*** = 50)	KALETRA group (*n* = 50)
**Age, years (SD)**^b^	**56.4 (16.3)**	**56.6 (17.8)**	**56.2 (14.8)**
**Sex, n (%)**	**100 (100)**	**50 (100)**	**50 (100)**
Male, n (%)	60 (60)	33 (66)	27 (54)
Female, n (%)	40 (40)	17 (34)	23 (46)
Marital status, n (%)	**100 (100)**	**50 (100)**	**50 (100)**
Single, n (%)	39 (39)	19 (38)	20 (40)
Married, n (%)	61 (61)	31 (62)	30 (60)
**Job, n (%)**	**90 (100)**	**44 (100)**	**46 (100)**
Hospital staff, n (%)	5 (5.5)	1 (2.3)	4 (8.7)
Self-employment, n (%)	32 (35.5)	16 (36.4)	16 (34.8)
Housekeeper, n (%)	35 (38.9)	15 (34.1)	20 (43.5)
Worker, n (%)	5 (5.6)	4 (9.1)	1 (2.2)
Employee, n (%)	13 (14.4)	8 (18.2)	5 (10.9)
**Smoker, n (%)**	15 (15)	12 (24)	3 (6)
**Cigarette per day, n (SD)**	12.2 (11.9)	13.5 (12.4)	5 (4.2)
**Smoking duration, years (SD)**	14.6 (8.2)	14.5 (8.6)	15 (7)
**Comorbidities, n (%)**	**72 (72)**	**39 (78)**	**33 (66)**
Hypertension, n (%)	39 (39)	22 (44)	17 (34)
Diabetes, n (%)	28 (28)	15 (30)	13 (26)
Coronary heart disease, n (%)	9 (9)	7 (14)	2 (4)
Asthma, n (%)	2 (2)	2 (4)	0 (0)
Chronic kidney disease, n (%)	2 (2)	1 (2)	1 (2)
Other diseases, n (%)	38 (38)	22 (44)	16 (32)

^a^Data is presented as mean (standard deviation), n (%) or n (SD); ^b^Abbreviation: *SD* standard deviation

In Table 2, clinical characteristics of the patients is shown. According to the signs and symptoms, patients in ARB group had weakness and headache more than the other group (70 and 26% versus 52 and 8%, respectively). About 6.7% of patients in KALETRA arm needed admission to Intensive Care Unit (ICU) versus 18.6% of ARB group. The duration of hospitalization in ARB group was significantly less than KALETRA arm (7.2 versus 9.6 days; *P* = 0.02). The severity of disease based on CT scan and chest X-ray findings were significantly different after 30 days of admission despite of almost similar severity at the day of admission. The mild finding based on CT scan was about 81% versus 53.2% in ARB and KALETRA groups, respectively. This result based on chest X-ray was 96% versus 67% in ARB and KALETRA arms, respectively.

**Table 2:** Clinical Characteristics of the Patients by Treatment Groups

Characteristics	All patients (*n* = 100)	Arbidol group (*n* = 50)	KALETRA group (*n* = 50)	***P*** Value
**Signs and Symptoms, n (%)**				
Fever	46 (46)	19 (38)	27 (54)	0.1
Cough	80 (80)	41 (82)	39 (78)	0.6
Shortness of breath	66 (66)	33 (66)	33 (66)	0.99
Weakness	61 (61)	35 (70)	26 (52)	0.6
Anosmia	10 (10)	6 (12)	4 (8)	0.5
Diarrhea	15 (15)	12 (24)	3 (6)	0.01
Nausea and vomiting	26 (26)	11 (22)	15 (30)	0.3
Myalgia	45 (45)	20 (40)	25 (50)	0.3
Headache	17 (17)	13 (26)	4 (8)	0.01
Others	50 (50)	18 (36)	32 (64)	0.005
**Hospitalization location, n (%)**	88 (100)	43 (100)	45 (100)	0.09
ICU, n (%)	43 (48.8)	8 (18.6)	3 (6.7)	
Ward, n (%)	45 (51.1)	35 (81.4)	42 (93.3)	
Hospitalization duration, days (SD)	8.4 (5.1)	7.2 (4.7)	9.6 (5.2)	0.02
Time to stop fever, days (SD)	2.9 (1.3)	2.7 (1.1)	3.1 (1.4)	0.2
**Illness severity, n (%)**				0.9
Mild	19 (19)	9 (18)	10 (20)	
Moderate	58 (58)	29 (58)	29 (58)	
Severe	23 (23)	12 (24)	11 (22)	
CT scan in beginning, n (%)	100 (100)	50 (100)	50 (100)	0.9
**Involvement type, n (%)**				
Mild	11 (11)	5 (10)	6 (12)	
Moderate	66 (66)	33 (66)	33 (66)	
severe	23 (23)	12 (24)	11 (22)	
CT scan in 30th day, n (%)	97 (100)	49 (100)	48 (100)	0.004
**Involvement type, n (%)**				
Mild	63 (64.9)	38 (80.9)	25 (53.2)	
Moderate	31 (31.9)	9 (19.1)	22 (46.8)	
severe	0 (0)	0 (0)	0	
CXR in beginning, n (%)	100 (100)	50 (100)	50 (100)	0.3
**Involvement type, n (%)**				
Mild	25 (25)	11 (22)	14 (28)	
Moderate	66 (66)	36 (72)	30 (60)	
severe	9 (9)	3 (6)	6 (12)	
CXR in 30th day, n (%)	97 (100)	49 (100)	48 (100)	< 0.001
**Involvement type, n (%)**				
Mild	75 (77.3)	44 (95.7)	31 (67.4)	
Moderate	17 (17.5)	2 (4.3)	15 (32.6)	
severe	0 (0)	0 (0)	0 (0)	
Intubation, n (%)	5 (5)	3 (6)	2 (4)	0.6
Mechanical Ventilation, n (%)	5 (5)	3 (6)	2 (4)	0.6
Mortality, n (%)	3 (3)	1 (2)	2 (4)	0.5
**Medication side effect, n (%)**	15 (100)	3 (100)	12 (100)	0.1
Nausea/vomiting, n (%)	8 (53.5)	1 (33.3)	7 (58.3)	
Dizziness, n (%)	3 (20)	0 (0)	3 (25)	
Others, n (%)	4 (26.7)	2 (66.7)	2 (16.7)	

Note: Data are presented as mean (standard deviation) or n (%)

Abbreviation: *ICU* Intensive Care Unit, *CT* Computerized Tomography, *CXR* Chest X-ray, *SD* Standard Deviation

Time to relief fever was similar across two groups (2.7 versus 3.1 days in ARB and KALETRA arms, respectively). Although the time for ARB group was less than KALETRA, but the difference was not statistically significant. The side effects of both drugs were not considerable. The most common adverse event was nausea and vomiting specially in KALETRA group. Patients in the ARB group experienced lower rates of nausea/vomiting compared to the KALETRA group. Among patients with nausea/vomiting about 33% versus 58% were belong to ARB and KALETRA respectively. Although this difference was not statistically significant. Furthermore, no evidence of dizziness was reported in the ARB group, while all patients with dizziness were from in KALETRA group. Compared to ARB, patients who received KALETRA had slightly higher ALT levels after 7 days of admission (32.2 ± 14.5 vs. 28.3 ± 15.6 U/L, respectively).

The intubation and need to mechanical ventilation rates were not different across two groups. Out of 100 patients, there were three deaths totally. One death occurred in ARB and two deaths in KALETRA group (Table 2).

According to laboratory findings, the conversion of CRP test was almost similar after 7 days of admission. Although, the two plus CRP was higher in patients in KALETRA group vs. ARB group (35%% versus 20%) but statistically insignificant (Table 3).

**Table 3:** Laboratory findings of the Patients by Treatment groups

Laboratory findings	All patients (*n* = 100)	Arbidol group (*n* = 50)	KALETRA group (*n* = 50)	***P*** Value
**CRP Base, n (%)**	100 (100)	50 (100)	50 (100)	0.6
0, n (%)	16 (16)	7 (14)	9 (18)	
+, n (%)	29 (29)	15 (30)	14 (28)	
++, n (%)	29 (29)	17 (34)	12 (24)	
+++, n (%)	26 (26)	11 (22)	15 (30)	
**CRP 7th day, n (%)**	99 (100)	50 (100)	49 (100)	0.2
0, n (%)	33 (33.3)	17 (34)	16 (32.7)	
+, n (%)	38 (38.3)	23 (46)	15 (30.6)	
++, n (%)	27 (27.2)	10 (20)	17 (34.7)	
+++, n (%)	1 (2)	0 (0)	1 (2)	
**PCR Base, n (%)**	100 (100)	50 (50)	50 (50)	0.8
Positive, n (%)	51 (51)	25 (50)	26 (52)	
**PCR Discharge, n (%)**	97 (100)	49 (100)	48 (100)	0.2
Positive, n (%)	18 (38.2)	7 (14.3)	11 (22.9)	
**Oxygen Saturation, % (SD)**				< 0.001
In admission	84.9 (8)	85.5 (8.4)	84.3 (7.7)	0.4
7th day	93 (4.2)	93.9 (3.1)	92 (4.8)	0.02
**Erythrocyte Sedimentation Rate, mm/h (SD)**				< 0.001
In admission	40 (19.9)	38.7 (19.7)	41.4 (20.3)	0.5
7th day	27.8 (17.4)	23.3 (15.5)	32.2 (18.2)	0.01
**White-cell count, ×10**^**9**^**/L (SD)**				< 0.001
In admission	10.1 (4.9)	10.5 (4.1)	9.8 (5.5)	0.4
7th day	6.7 (2.2)	6.2 (1.7)	7.2 (2.5)	0.03
**Lymphocyte count, × 10**^**9**^**/L (SD)**				< 0.001
In admission	20.4 (8.7)	20.3 (8.7)	20.4 (8.9)	0.9
7th day	26.3 (10.6)	24.7 (8.9)	27.9 (12)	0.1
**Neutrophil count, ×10**^**9**^**/L (SD)**				< 0.001
In admission	73.8 (11.2)	74.6 (9.7)	73 (12.6)	0.4
7th day	65.9 (13.2)	69.1 (11.2)	62.7 (14.4)	0.01
**Neutrophil/ Lymphocyte ratio (SD)**				< 0.001
In admission	4.7 (3.3)	4.8 (3.7)	4.6 (2.8)	0.7
7th day	3.4 (3.7)	3.7 (4.3)	3.2 (2.9)	0.4
**AST, IU/L (SD)**				0.9
In admission	34.3 (19.9)	33.8 (23.7)	34.7 (15.5)	0.8
7th day	32.5 (15.2)	31.1 (15.9)	33.8 (14.4)	0.3
**ALT, IU/L (SD)**				0.1
In admission	28.8 (16.4)	28.1 (18.2)	29.5 (14.6)	0.6
7th day	30.2 (15.1)	28.3 (15.6)	32.2 (14.5)	0.2
**Total Bilirubin, mg/dL (SD)**				0.3
In admission	0.9 (0.5)	1 (0.5)	0.9 (0.4)	0.3
7th day	1 (0.4)	1 (0.5)	1 (0.4)	0.8
**Serum Creatinine,** μmol**/L (SD)**				0.01
In admission	1 (0.6)	1.1 (0.8)	0.9 (0.2)	0.1
7th day	0.9 (0.4)	1 (0.6)	0.9 (0.2)	0.1
**Blood Sodium, mEq/L (SD)**				< 0.001
In admission	136.3 (3.7)	136.1 (3.8)	136.4 (3.6)	0.7
7th day	140.5 (3)	140.7 (2.9)	140.3 (3.1)	0.5
**Blood Potassium, mmol/L (SD)**				0.08
In admission	3.9 (0.5)	3.9 (0.5)	3.9 (0.5)	0.9
7th day	4 (0.5)	3.9 (0.4)	4.2 (0.5)	0.001
**TSH, mIU/L (SD)**				0.3
In admission	4.2 (1.8)	4.4 (1.9)	4.1 (1.7)	0.3
7th day	4.1 (1.8)	4.2 (1.9)	4 (1.7)	0.5

Note: Data are presented as mean (standard deviation) or n (%)

Abbreviation: *CRP* C-Reactive Protein, *PCR* Polymerase Chain Reaction, *SD* Standard Deviation, *U/L* Units/Liter, *mm/h* Millimeter/Hour, *SGOT* Serum Glutamic Oxaloacetic Transaminase, *SGPT* Serum Glutamic-Pyruvic Transaminase, *mg/dl* Milligrams per Deciliter, *TSH* Thyroid Stimulating Hormone

*P-values indicate differences between patients in the Arbidol and the KALETRA groups and between in hospital and 7th day laboratory findings. P < 0.05 was considered statistically significant

The PCR of 50% of patients at the time of admission was positive. This proportion at the time of discharge from hospital was about 38% totally. The positive PCR rate at the time of discharge in KALETRA and ARB group was 23 and 14%, respectively (Table 3).

Peripheral oxygen saturation rate was significantly different after 7 days of admission across two groups (94% versus 92% in ARB and KALETRA groups, respectively) (*P* = 0.02). Also WBC and neutrophil counts, ESR and blood K were significantly different after 7 days of admission between ARB and KALETRA arms despite of similar values at the time of admission. Totally apart from comparative groups, peripheral oxygen saturation, ESR, WBC, neutrophil and lymphocyte counts, neutrophil to lymphocyte ratio, and blood Na were significantly different at the time of admission and 7 days after significantly (*P* < 0.001) (Table 3).

Patients who had history of ischemic heart disease (IHD) were hospitalized more than the patients without this history (11.3 (4.9) versus 8.1 (5.0) days). This difference was statistically significant at less than 0.1 (*P* = 0.09) partially. The duration of hospitalization for patients with diabetes mellitus (DM) was also more than patients without this disease (10 (4.0) versus 7.8 (5.3) days). This difference was statistically significant (*P* = 0.04). Based on CT scan findings at the time of admission, the patients who categorized in severe group hospitalized more days than the mild group. (12 (5.4) versus 2 (2.0) days) (*P* < 0.01). Patients with more peripheral saturation of oxygen had a significantly shorter duration of hospitalization than the patients with lower saturation (r = 0.50; *P* = 0.01). High WBC count at the time of admission was correlated with higher duration of hospitalization (r = 0.27; *P* = 0.007). The Na level and lymphocytosis at the time of admission were correlated with duration of hospitalization reversely (r = − 0.32; *P* = 0.01, r = − 0.15; *P* = 0.05, respectively) (data was not shown). The laboratory data were not different based on severity of disease at the time of admission, but WBC count was higher in severe cases than mild and moderate categories (15 versus 8.8 and 8.1 × 10^9^/L, respectively).

Based on multiple linear regression analysis, IHD, Na level and oxygen saturation at the time of admission and type of therapy were the independent adjusted variables that determined the duration of hospitalization in patients with COVID-19. The lymphocytosis at the level of 0.06 probability value could be another determinant factor for duration of hospitalization (Table 4).

**Table 4:** Regression analysis to determine factors associated with duration of hospitalization

Variables^a^		B	Std. Error	t	***P*** Value	95.0% Confidence Interval for B	
						Lower Bound	Upper Bound
	(Constant)	73.438	15.261	4.812	.000	43.129	103.747
	Arbidol group	2.596	.837	3.102	.003	.934	4.258
	Without IHD^b^	−3.842	1.482	−2.593	.011	−6.786	−.899
	Without DM^b^	.122	1.005	.121	.904	−1.874	2.118
	WBC	.113	.093	1.214	.228	−.072	.298
	Oxygen saturation	−.238	.061	−3.900	.000	−.359	−.117
	Lymphocytosis	−.089	.048	−1.865	.065	−.183	.006
	Na level	−.300	.112	−2.670	.009	−.523	−.077

^a^The White blood cell count (WBC), lymphocytosis, Na level and Oxygen saturation are for the time of admission

^b^Ischemic heart disease; Diabetes Mellitus

## Discussion

Since COVID-19 was became a pandemic, a variety of antiviral drugs have been investigated on patients with COVID-19 [13]. Arbidol is a Russian antiviral drug that seems to be effective against many viruses including influenza A, B, and C, respiratory syncytial virus (RSV), severe acute respiratory syndrome-related coronavirus (SARS-CoV), adenovirus, parainfluenza, poliovirus, rhinovirus, coxsackievirus, Zika virus, hepatitis B and C viruses [14–16]. It has been demonstrated that ARB has a dual effect on cell attachment and replication, and thus a broad-spectrum effect on viruses [8, 17], so it is administered for post-exposure prophylaxis and treatment [18]. Therefore, ARB is considered to be one of the antiviral drugs that can be effective in the treatment of COVID-19 patients. In the present randomized controlled trial, we compared the efficacy and safety of antiviral ARB to KALETRA in COVID-19 patients and showed several benefits in the ARB group compared to KALETRA group. During the study, 100 patients were assigned, 50 patients were assigned to receive ARB and 50 to receive KALETRA.

We reported that nausea/vomiting was the most common side effect among the study participants. Data also showed that both drugs had no seriously side effects. Similarly, reports found no life-threatening adverse events in ARB and KALETRA groups [19, 20], except Li et al. study, which presented an old male patient with a history of diabetes mellitus and hypertension in the KALETRA group, who experienced severe diarrhea on day three of initiating treatment [19].

COVID-19 Patients treated with KALETRA are more likely to show higher WBC counts and CRP serum levels than those who received ARB. Regarding WBC differential, neutrophil counts seem to be higher, while lymphocyte counts appear to be lower in KALETRA-treated patients than in ARB [20]. In the present report, we observed that KALETRA, compared to ARB, was significantly associated with higher counts of WBC and ESR serum levels after 7 days of admission. On the other hand, although we found that patients in the KALETRA group had higher proportions of CRP ≥ 2+ than the ARB group 7 days after admission, there was no significant difference between them. After 7 days of admission, neutrophil counts were significantly lower in the KALETRA group than in ARB. However, different findings between reports could be attributed to differences in the design of studies, treatment regimens, and sample sizes.

We have shown that slightly higher proportions of patients in the ARB group had severe clinical status compared to the KALETRA group (24% vs. 22%, respectively), while it did not significantly differ between groups. However, ARB is considered to minimize the rates of worsening of the clinical condition. It has been reported that a lower prevalence of COVID-19 patients who received ARB (8.6%) deteriorated to severe clinical status compared to patients who received KALETRA (23.5%) during the hospitalization, but no significant difference between the two groups of treatment was found [19].

Although all of the patients in both groups had similar severity on admission, 18.6% of patients in ARB group were candidates for referring to the intensive care unit (ICU) during admission versus 81% of patients in KALETRA group. Patients in the ARB group spent a shorter duration of hospitalization (7.2 days) compared to KALETRA group (9.6 days, *p*-value = 0.02). Furthermore, 81% of patients in ARB group had mild involvement on the chest CT scan after 30 days of admission compared to 53% in KALETRA group (*p*-value = 0.004). Noteworthy, we noticed several demographic, clinical, and laboratory determinants of duration of hospitalization in COVID-19 patients, including IHD, oxygen saturation on admission, treatment with ARB, plasma Na levels, and lymphocytosis with a probability value of 0.06. In this regard, two cohort studies from Wuhan, China noted that COVID-19 patients aged ≥80 years and with lymphopenia (< 1.1 × 10^9^/L) had a longer duration of hospitalization [21, 22].

Collectively, our findings indicate a lower proportion of ICU admissions, a shorter length of hospital stay, and a higher percentage of mild chest CT involvement after 30 days of admission among the COVID-19 patients who were treated with ARB compared to KALETRA, representing that ARB may be superior to KALETRA in the management of COVID-19 patients. Although, to date, no vaccines or antiviral drugs are approved for the treatment of COVID-19, “National Health Commission and National Administration of Traditional Chinese Medicine” have recently recommended KALETRA combined with ARB and reported its antiviral effects [23]. However, to our knowledge, limited documents are evaluating the efficacy and safety of ARB on COVID-19 patients. Consistent with our study, a retrospective study from China compared the efficacy and safety of KALETRA to ARB in COVID-19 patients [20]. They detected no viral load in the ARB group, while a viral load of 44.1% was found in the KALETRA group, concluded that ARB monotherapy might be more effective than KALETRA for COVID-19 treatment. Similarly, another retrospective study found that ARB, combined with KALETRA, compared to KALETRA alone, would improve the viral clearance and chest CT scans [24]. A cohort study of 504 hospitalized COVID-19 patients with mixed illness severities presented that ARB significantly reduced mortality (OR = 0.183, 95% CI = 0.075–0.446), and it was more likely to absorb lesions on chest CT scan [25]. However, a randomized controlled trial by Li et al. and a retrospective study by Chen et al. suggested that neither the COVID-19 symptoms or chest CT involvement, nor the time to SARS-CoV-2 PCR negative in respiratory specimens was improved/decreased in patients who received KALETRA and ARB [19, 26]. Despite the small sample size in Li et al. study [19], they did not recruit severely or critically ill cases. Additionally, different from Li et al. [19], we gave KALETRA and ARB in combination with hydroxychloroquine to each group. Nevertheless, we believe that our findings are likely to help physicians develop appropriate treatment strategies among evolving evidence for COVID-19 management.

## Conclusion

To the best of our knowledge, the present randomized controlled trial is the first study from Iran, highlighting the benefits of ARB monotherapy for the treatment of hospitalized COVID-19 patients. We have shown that ARB, compared to KALETRA, significantly contributes to clinical and laboratory improvements, including peripheral oxygen saturation, requiring ICU admissions, duration of hospitalization, chest CT involvements, WBC, and ESR.

## Data Availability

The datasets during and/or analyzed during the current study available from the corresponding author on reasonable request.

## Abbreviations

COVID-19: Corona Virus Disease 19
Arbidol: ARB
PHELC: Public Health Emergency of International Concern
SARS-Cov-2: Severe Acute Respiratory Syndrome Corona virus 2
WHO: World Health Organization
HCV: Hepatitis C Virus
IUMS: Iran University of Medical Sciences
CRP: C-reactive protein
CBC: Complete Blood Cell Count
ESR: Erythrocyte Sedimentation Rate
AST: Aspartate transaminase
ALT: Alanine aminotransferase
WBC: White Blood Cell Count
TSH: Thyroid Stimulating Hormone
IHD: Ischemic heart disease
RT-PCR: Reverse Transcriptase Polymerase Chain Reaction
CT: Computed Tomography
IRCT: Iranian Registry of Clinical Trials
CXR: Chest X ray
ANOVA: Analysis of Variance
TDS: Three times daily
BD: Twice daily
SPSS: Statistical Software for Social Sciences

## References

[1] World Health Organization. Corona virus overview. WHO. Available at: https://www.who.int/health-topics/coronavirus#tab=tab_1 . Accessed on 4/5/2020.

[2] Worldometers. COVID-19 CORONAVIRUS PANDEMIC. Available at: https://www.worldometers.info/coronavirus/ . Accessed 8 Aug 2020.

[3] World Health Organization Europe 2019-nCoV outbreak is an emergency of international concern: httsp://www.euro.who.int/en/health-topics/health emergencies/internationalhealth-regulations/news/news/2020/2/2019-ncov-outbreakis-an-emergency-of-international-concern; (Accessed February 22, 2020).

[4] Xiao Y, Torok ME (2020). Taking the right measures to control COVID-19. Lancet Infect Dis.

[5] Al-Tawfiq JA, Al-Homoud AH, Memish ZA. Remdesivir as a possible therapeutic option for the COVID-19. Travel Med Infect Dis. 2020;34:101615. 10.1016/j.tmaid.2020.101615 PMC7129391 32145386

[6] Gao J, Tian Z, Yang X. Breakthrough: Chloroquine phosphate has shown apparent efficacy in treatment of COVID-19 associated pneumonia in clinical studies. Bioscience Trends. 2020. 10.5582/bst.2020.01047. 10.5582/bst.2020.01047 32074550

[7] Caly L, Druce JD, Catton MG, Jans DA, Wagstaff KM. The FDA-approved Drug Ivermectin inhibits the replication of SARS-CoV-2 in vitro. Antivir Res. 10.1016/j.antiviral.2020.104787. 10.1016/j.antiviral.2020.104787 PMC7129059 32251768

[8] Boriskin YS, Leneva IA, Pécheur EI, Polyak SJ (2008). Arbidol: a broad-spectrum antiviral compound that blocks viral fusion. Curr Med Chem.

[9] Pécheur E-I, Borisevich V, Halfmann P, Morrey JD, Smee DF, Prichard M (2016). The synthetic antiviral drug Arbidol inhibits globally prevalent pathogenic viruses. J Virol.

[10] Zheng LU, Zhang L, Huang J, Nandakumar KS, Liu S, Cheng K (2020). Potential treatment methods targeting 2019-nCoV infection. Eur J Med Chem.

[11] Bernheim A , Mei X, Huang M, Yang Y, Fayad ZA, Zhang N, et al. Chest CT Findings in Coronavirus Disease-19 (COVID-19): Relationship to Duration of Infection. Feb 20 2020 10.1148/radiol.2020200463. 10.1148/radiol.2020200463 PMC7233369 32077789

[12] Campbell MJ, Machin D, Walters SJ (2007). Medical Statistics: A Textbook for the Health Sciences Wiley, 4th Edition.

[13] Amawi H, Abu Deiab GI, AA DK, Tambuwala MM (2020). COVID-19 pandemic: an overview of epidemiology, pathogenesis, diagnostics and potential vaccines and therapeutics. Ther Deliv.

[14] Hulseberg CE, Fénéant L, Szymańska-de Wijs KM, Kessler NP, Nelson EA, Shoemaker CJ, et al. Arbidol and other low-molecular-weight drugs that inhibit lassa and ebola viruses. J Virol. 2019;93(8):e02185-18. 10.1128/JVI.02185-18 PMC6450122 30700611

[15] Fink SL, Vojtech L, Wagoner J, Slivinski NSJ, Jackson KJ, Wang R (2018). The antiviral drug Arbidol inhibits Zika virus. Sci Rep.

[16] Leneva IA, Falynskova IN, Makhmudova NR, Poromov AA, Yatsyshina SB, Maleev VV (2019). Umifenovir susceptibility monitoring and characterization of influenza viruses isolated during ARBITR clinical study. J Med Virol.

[17] Blaising J, Polyak SJ, Pécheur E-I (2014). Arbidol as a broad-spectrum antiviral: an update. Antivir Res.

[18] Zhang JN, Wang WJ, Peng B, Peng W, Zhang YS, Wang YL (2020). Potential of Arbidol for post-exposure prophylaxis of COVID-19 transmission: a preliminary report of a retrospective cohort study. Curr Med Sci.

[19] Li Y, Xie Z, Lin W, Cai W, Wen C, Guan Y, et al. An exploratory randomized, controlled study on the efficacy and safety of lopinavir/ritonavir or arbidol treating adult patients hospitalized with mild/moderate COVID-19 (ELACOI). MedRxiv. 2020.

[20] Zhu Z, Lu Z, Xu T, Chen C, Yang G, Zha T (2020). Arbidol monotherapy is superior to lopinavir/ritonavir in treating COVID-19. J Infect.

[21] Zheng H, Tan J, Zhang X, Luo A, Wang L, Zhu W, et al. Impact of sex and age on respiratory support and length of hospital stay among 1792 patients with COVID-19 in Wuhan, China. BJA. 2020. 10.1016/j.bja.2020.07.001. 10.1016/j.bja.2020.07.001 PMC7365067 32773217

[22] Liu X, Zhou H, Zhou Y, Wu X, Zhao Y, Lu Y (2020). Risk factors associated with disease severity and length of hospital stay in COVID-19 patients. J Infect.

[23] Xu K, Cai H, Shen Y, Ni Q, Chen Y, Hu S (2020). Management of corona virus disease-19 (COVID-19): the Zhejiang experience. Zhejiang da xue bao Yi xue ban.

[24] Deng L, Li C, Zeng Q, Liu X, Li X, Zhang H (2020). Arbidol combined with LPV/r versus LPV/r alone against Corona virus disease 2019: a retrospective cohort study. J Infect.

[25] Liu Q, Fang X, Tian L, Chen X, Chung U, Wang K, et al. The effect of Arbidol Hydrochloride on reducing mortality of COVID-19 patients: a retrospective study of real world date from three hospitals in Wuhan. MedRxiv. 2020. 10.1101/2020.04.11.20056523.

[26] Chen J, Ling Y, Xi X, Liu P, Li F, Li T, et al. Efficacies of lopinavir/ritonavir and arbidol in the treatment of novel coronavirus pneumonia. Chin J Infect Dis. 2020;38(0):E008.

